# Statement of Retraction: MYCN contributes to the sensitization of acute myelogenous leukemia cells to cisplatin by targeting SRY-box transcription factor 4

**DOI:** 10.1080/21655979.2024.2302650

**Published:** 2024-02-01

The Publisher of the journal *Bioengineered*, have retracted the following article:

Xianbao Huang, Ling Qi, Wei Lu, Ziye Li, Wuping Li and Fei Li. MYCN contributes to the sensitization of acute myelogenous leukemia cells to cisplatin by targeting SRY-box transcription factor 4. Bioengineered. 2021 Oct. doi: 10.1080/21655979.2021.1997697.

Since publication, significant concerns have been raised about the compliance with ethical policies for human research and the integrity of the data reported in the article.

When approached for an explanation, the authors provided some original data but were not able to provide all the necessary supporting information. As verifying the validity of published work is core to the scholarly record’s integrity, we are retracting the article. All authors listed in this publication have been informed.

We have been informed in our decision-making by our editorial policies and the COPE guidelines.

The retracted article will remain online to maintain the scholarly record, but it will be digitally watermarked on each page as ‘Retracted.’

